# Self-Reported Knowledge, Attitudes, Perceptions, and Readiness Regarding AI Among Obstetrics and Gynecology Trainees: Cross-Sectional Study

**DOI:** 10.2196/97072

**Published:** 2026-09-10

**Authors:** Maya Alazrae’i, Ismaiel Abu Mahfouz, Zain Al-sarayreh, Jawad Jraisat, Fatima alzahra Abo Abood, Nabil Nwairan, Rakan Lallas

**Affiliations:** 1 Jordan Hospital, Amman, Amman, Jordan; 2 Al-Balqa Applied University, Al Salt, Jordan. P O Box 19117, Al-Salt, Jordan, Al Salt, Balqa, 19117, Jordan, 962 53491111; 3 Al Hussain Al Salt New Hospital, Al Salt, Al Salt, Jordan

**Keywords:** artificial intelligence, AI, trainees, knowledge, attitude, perception, obstetrics and gynecology

## Abstract

**Background:**

AI technologies refer to computer-based systems designed to perform tasks that typically require human intelligence and have been increasingly used in obstetrics and gynecology (O&G).

**Objective:**

This study aimed to assess O&G trainees’ self-reported knowledge of AI, attitude toward its introduction into clinical practice, perception of its clinical importance, and their readiness for its introduction.

**Methods:**

A cross-sectional study was conducted from December 1, 2024, to December 31, 2024, among O&G trainees in Jordan. Data were collected on participants’ characteristics, self-reported knowledge of AI in O&G, their attitudes toward its introduction, and perception of its importance. The scores of the 3 domains and the study-specific knowledge, attitude, and perception (KAP)–based readiness were converted into percentages of their maximum attainable scores and were grouped into low, moderate, and high categories. Multivariable linear regression analysis was used to identify variables associated with KAP-based readiness.

**Results:**

A total of 218 trainees were recruited; the median age was 28 (IQR 24-38) years, 180 (82%) participants were female, 117 (53.7%) were junior trainees, 148 (67.9%) were working in public hospitals, 183 (83.9%) reported “average or better” knowledge of IT, and 196 (89.9%) had never received formal training on the medical applications of AI. The highest median percentage score was for self-reported knowledge (71.1%, IQR 62.2%‐75.6%). Additionally, KAP-based readiness was moderate in 188 (86.2%) participants. In multivariable linear regression, none of the examined trainee characteristics were independently associated with the KAP-based readiness (all *P* values >.05).

**Conclusions:**

O&G trainees in Jordan demonstrated moderate self-reported knowledge of AI, generally positive attitudes and perceptions of its importance, and moderate study-specific KAP-based readiness. Moreover, formal training on AI medical applications was uncommon, and most trainees supported the integration of AI training into medical education. These findings support the need for structured AI education, and future research should evaluate broader and objectively measured individual and organizational determinants of AI-related readiness.

## Introduction

AI technologies enable computers and machines to simulate human abilities in learning, problem-solving, decision-making, and creativity [1]. These technologies have been increasingly introduced in various medical specialties [1], such as the use of deep learning for the analysis of medical images, the application of natural language processing for electronic health records, and decision support systems to aid clinical decision-making [2]. Moreover, AI medical technologies are expected to transform health care practices and may change physicians’ duties and responsibilities [3].

The use of AI technologies in obstetrics and gynecology (O&G) is increasing. In maternal or fetal medicine, AI may help identify women at higher risk of pregnancy complications, such as preeclampsia [4], and has potential applications in prenatal screening and the diagnosis of congenital anomalies [5]. Intrapartum applications include the analysis and interpretation of fetal heart rate tracings and events, which may enhance the quality of intrapartum care [6]. Furthermore, in reproductive endocrinology, AI-assisted algorithms help optimize the management of ovarian stimulation and improve clinical decision-making during in vitro fertilization [7]. In gynecology oncology, AI has improved the early detection of cervical cancer through the analysis of Pap smears and colposcopy images, while also enabling more personalized treatment approaches for ovarian and endometrial cancers [8].

A recent report showed that while around three-quarters of intern doctors have poor knowledge of AI, the majority perceived it positively [9]. In O&G training, Desseauve et al [10] showed a similar pattern and reported that O&G trainees have low knowledge in IT and AI, in addition to a discrepancy between self-reported and objectively measured AI proficiencies. Moreover, Tolentino et al [11] showed that current AI education across all stages of medical training, including students, trainees, and practicing physicians, is fragmented and lacks standardized curriculum frameworks. These findings support the need for structured AI training programs.

In health care, readiness for AI technologies refers to the extent to which health care professionals and organizations are willing and able to integrate them into clinical practice [12]. From the perspective of health care professionals, readiness involves relevant knowledge and skills, favorable attitudes toward AI, recognition of its potential importance, and awareness of its limitations and ethical implications. At the organizational level, this includes relevant infrastructure, training, governance, and technical support [13].

Limited evidence exists regarding O&G trainees’ AI-related knowledge, their perception of its importance, and their attitudes toward its introduction to clinical practice. The availability of such data may support postgraduate training programs directors in the development of targeted AI educational initiatives. Therefore, this study aimed to assess O&G trainees’ self-reported AI knowledge, attitude, and perception (KAP) and their KAP-based readiness.

## Methods

### Study Type, Sites, and Population

A cross-sectional, self-administered questionnaire survey was conducted between December 1, 2024, and December 31, 2024. All public and private hospitals in Jordan that offer O&G training programs were included. Trainees holding a professional degree in IT were excluded.

### Study Instrument

An English-language hard copy questionnaire was developed by the researchers and was informed by relevant published literature on AI technologies [14-18]. Additionally, ChatGPT was used to suggest some questionnaire items. The research team reviewed these suggestions for relevance and clarity and decided which items to retain, edit, or exclude from the final version of the questionnaire. Content validity was established by 3 gynecologists. Clarity and comprehension were assessed by the results of a pilot study that included 20 trainees [19]. All comments were considered in the final version of the questionnaire that was used for data collection. Moreover, the data collected from the pilot study were not included in the final analysis because the questionnaire was subsequently modified.

The questionnaire consisted of 2 parts. The first part collected data on the participants’ characteristics, including age, gender, residency year, place of training (public or private hospital), and number of years of experience since graduation (fresh graduate: <3 years; senior graduate: ≥3 years). In addition, the participants’ current self-reported knowledge of IT was measured using a 5-point Likert scale (very poor, below average, average, above average, or excellent). Furthermore, participants were asked about their knowledge sources of the medical applications of AI (formal lecture, conference, internet, or a colleague), if they ever had formal training on the medical applications of AI during residency training (yes, no, or not sure), and if they believe that AI should be included in undergraduate and postgraduate medical training (yes, no, or not sure).

The second part of the questionnaire was used to assess trainees’ self-reported knowledge about current applications of AI in O&G, their attitudes toward its introduction into clinical practice, and their perceptions of importance. This part comprised 36 statements across 3 domains: 9 assessing self-reported knowledge, 21 assessing attitudes, and 6 assessing perceptions. Moreover, the unequal number of items in each domain was not intentional but resulted from the number of items generated for each domain during questionnaire development. Of the 36 items, 21 (58.3%) assessed attitude, 9 (25%) assessed self-reported knowledge, and 6 (16.7%) assessed perception. At the beginning of each domain, candidates were asked the following question: “How much do you agree with the following statements?” This was followed by a series of statements to which participants responded using a 5-point Likert scale (strongly agree, agree, undecided, disagree, and strongly disagree). The statements were phrased to reflect high self-reported knowledge, positive attitude, and positive perception of importance (Multimedia Appendix 1).

### Study Procedure

Members of the research team approached potential participants in the research sites during the morning report meetings. This time was chosen because it was when most members of the team, including those who had been on call the previous day, those on call that day, and trainees assigned to other duties, were expected to be present. The research objectives and the inclusion and exclusion criteria were explained to the participants.

### Sample Size Calculation

The total number of O&G trainees in Jordan at the time of the study was 497. Therefore, a sample size of 217 was required to achieve a 95% confidence level and a 5% margin of error. Allowing for a 20% rate of ineligible responses, we aimed to recruit 270 participants for the study [20].

### Statistical Analysis

Several variables were grouped for better comparisons. These included age in years (<30 and ≥30 years), residency year (junior: years 1 and 2 and senior: years 3, 4, and 5), number of years since graduation (junior graduate: <3 years and senior graduate: ≥3 years), place of training (public or private hospital), current self-reported knowledge of AI and medical applications of AI (below average or poorer, and average or better), and source of knowledge about the medical application of AI (internet or noninternet).

For the calculation of self-reported KAP domain scores, the Likert scale responses were converted into numerical values (strongly agree=5, agree=4, undecided=3, disagree=2, and strongly disagree=1). Then, the maximum attainable scores for each domain were calculated by summing the responses, with maximum scores of 45, 105, and 30 for the self-reported knowledge, attitude, and perception domains, respectively. Additionally, the maximum attainable scores of the 3 domains were summed to generate a study-specific KAP score, with a maximum possible score of 180. In this study, the KAP score was used to define KAP-based readiness.

To allow comparisons across the scores of the 3 domains and the KAP-based readiness, scores were converted into percentages of their respective maximum attainable scores. For descriptive purposes, the total scores of the 3 domains were further grouped using the modified Bloom cut-off points: <60%, 60% to 79%, and ≥80%. Therefore, self-reported knowledge, attitude, perception, and KAP-based readiness scores were grouped as low, moderate, or high [21].

Data normality was tested using the Shapiro-Wilk test. For normally distributed data, descriptive statistics were reported as mean (SD), whereas nonparametric and Likert scale data were reported as median (IQR). Spearman rank correlation was used to study the relationships between the percentage scores of the 3 domains. Bivariable analyses were conducted to examine associations between KAP-based readiness categories and participant characteristics. Additionally, multivariable linear regression analysis was performed using the KAP-based readiness percentage score as a continuous variable. Age group, gender, residency year, place of training, self-reported IT knowledge, source of AI knowledge, and formal AI training during residency were entered simultaneously as independent variables. Multicollinearity was assessed, and sensitivity analyses were performed.

### Missing Data

A total of 270 questionnaires were distributed, and all were returned. Of these, 52 (19.3%) were excluded because they had more than 20% missing item-level data. The remaining 218 questionnaires were included in the final analysis. Among the included questionnaires, missing data were <3% and were replaced using the median or modes, as appropriate [22].

Data were analyzed using SPSS (version 26; IBM Corp). The level of significance was set at α<.05.

### Ethical Considerations

Ethics approval was granted by the research committee of the Faculty of Medicine, Al-Balqa Applied University (reference number: ب ع ط / 2024 / 25). In reporting this study, we followed STROBE (Strengthening the Reporting of Observational Studies in Epidemiology) [23] and KAP studies [24] reporting guidelines. Participants were informed that no personal identifiable information would be collected, no incentives would be offered to participate in the study, and the mean time needed for the completion of the questionnaire was 7 minutes. Verbal rather than written consents were obtained because of the limited time available during morning report meetings. Members of the research team explained the study aims and the voluntary nature of participation. Questionnaires were distributed to trainees who verbally agreed to participate.

## Results

### Participants’ Characteristics

Of the 218 trainees recruited, the median age was 28 (IQR 24-38) years, 180 (82.6%) participants were female, 117 (53.7%) were junior trainees, 128 (58.7%) were senior graduates, and 148 (67.9%) reported training at a public hospital. Additionally, “average or better” self-reported IT knowledge was expressed by 183 (83.9%) participants. The internet was the most commonly reported source of knowledge about the medical applications of AI, reported by 165 (75.7%) participants, and 151 (69.3%) and 160 (73.4%) participants believed that AI training should be introduced to undergraduate and postgraduate medical education, respectively (Table 1).

**Table 1.** Participants’ characteristics.

Variables	Participants
Age (years), median (IQR)	28 (24-38)
Age group (years), n (%)	
24-29	155 (71.1)
≥30	63 (28.9)
Gender, n (%)	
Man	38 (17.4)
Woman	180 (82.6)
Residency year, n (%)	
Junior trainee (years 1-2)	117 (53.7)
Senior trainee (years 3-5)	101 (46.3)
Years since graduation from medical school, median (IQR)	4 (1-12)
Years since graduation from medical school, n (%)	
Junior graduate (<3)	90 (41.3)
Senior graduate (≥3)	128 (58.7)
Place of training, n (%)	
Public hospital	148 (67.9)
Private hospital	70 (32.1)
Self-reported knowledge of IT, n (%)	
Below average or poorer	35 (16.1)
Average or better	183 (83.9)
Knowledge sources about medical applications of AI, n (%)	
Internet	165 (75.7)
Noninternet	53 (24.3)
Had formal training on the medical applications of AI during training, n (%)	
Yes	22 (10.1)
No	196 (89.9)
Belief that AI should be included in undergraduate medical education, n (%)	
Yes	151 (69.3)
No or not sure	67 (30.7)
Belief that AI should be included in postgraduate medical education, n (%)	
Yes	160 (73.4)
No or not sure	58 (26.6)

### Self-Reported KAP and KAP-Based Readiness

The results showed that Cronbach α was 0.79 for self-reported knowledge, 0.82 for attitudes, 0.71 for perceptions, and 0.83 for study-specific KAP-based readiness, indicating acceptable to good internal consistency. Data analysis showed statistically significant positive correlations between self-reported knowledge and attitude (*r*=0.39; 95% CI 0.26-0.51; *P*<.001), self-reported knowledge and perception of importance (*r*=0.47; 95% CI 0.34-0.58; *P*<.001), and attitude and perception of importance (*r*=0.59; 95% CI 0.47-0.68; *P*<.001).

Table 2 shows the median (IQR) of the percentages of the maximum attainable scores of the 3 domains and the KAP-based readiness, in addition to the 3 modified Bloom’s categories of the respective domains. The analysis showed that the moderate category was most frequent in all domains: self-reported knowledge: 72.9% (159/218); attitude: 80.7% (176/218); and perception: 65.6% (143/218).

**Table 2.** Distribution and categorization of domain-specific and total study–specific knowledge, attitude, and perception (KAP)–based readiness scores (N=218)^a^.

Domains and modified Bloom’s categories	Percentage of the maximum attainable scores, median (IQR)	Participants, n (%)
Self-reported knowledge	71.1 (62.2‐75.6)	
Low		21 (9.6)
Moderate		159 (72.9)
High		38 (17.4)
Attitude	68.6 (62.6‐74.3)	
Low		26 (11.9)
Moderate		176 (80.7)
High		16 (7.3)
Perception	70.0 (63.3‐76.7)	
Low		29 (13.3)
Moderate		143 (65.6)
High		46 (21.1)
Study-specific KAP-based readiness	69.4 (63.9‐73.3)	
Low		18 (8.3)
Moderate		188 (86.2)
High		12 (5.5)

a Categories were based on modified Bloom’s cut-off points (low: <60%; moderate: 60%-79%; and high: >80%).

The KAP-based readiness was moderate in 86.2% (188/218) of the participants. The distribution of the 3 KAP-based readiness categories in different subgroups of participants’ characteristics is shown in Table 3. Moderate KAP-based categories were the most common in every subgroup, ranging from 81.8% (18/22) to 97.1% (34/35). While low KAP-based readiness was highest among participants who trained at private hospitals (10/70, 14.3%), high KAP-based readiness was highest among participants who had received formal AI training during residency (3/22, 13.6%).

**Table 3.** Distribution of study-specific knowledge, attitude, and perception (KAP)–based readiness across the different subgroups of participants’ characteristics (N=218).

Variables and categories	Low KAP, n (%)	Moderate KAP, n (%)	High KAP, n (%)
Age group (years)			
<30	13 (8.4)	133 (85.8)	9 (5.8)
≥30	5 (7.9)	55 (87.3)	3 (4.8)
Gender			
Man	2 (5.3)	35 (92.1)	1 (2.6)
Woman	16 (8.9)	153 (85)	11 (6.1)
Residency year			
Junior resident (years 1-2)	10 (8.5)	100 (85.5)	7 (6)
Senior resident (years 3‐5)	8 (7.9)	88 (87.1)	5 (5)
Years since graduation			
Fresh graduate (<3 years)	8 (8.9)	78 (86.7)	4 (4.4)
Senior graduate (≥3 years)	10 (7.8)	110 (85.9)	8 (6.3)
Place of training			
Public hospital	8 (5.4)	130 (87.8)	10 (6.8)
Private hospital	10 (14.3)	58 (82.9)	2 (2.9)
Self-reported IT knowledge			
Below average or poorer	0 (0)	34 (97.1)	1 (2.9)
Average or better	18 (9.8)	154 (84.2)	11 (6)
Source of AI knowledge			
Internet	16 (9.7)	139 (84.2)	10 (6.1)
Noninternet source	2 (3.8)	49 (92.5)	2 (3.8)
Formal AI training during residency			
Yes	1 (4.5)	18 (81.8)	3 (13.6)
No	17 (8.7)	170 (86.7)	9 (4.6)
Belief that AI should be included in undergraduate training			
Yes	10 (6.6)	131 (86.8)	10 (6.6)
No or not sure	8 (11.9)	57 (85.1)	2 (3)
Belief that AI should be included in postgraduate training			
Yes	10 (6.3)	139 (86.9)	11 (6.9)
No or not sure	8 (13.8)	49 (84.5)	1 (1.7)

### Associations With Study-Specific KAB-Based Readiness

In the bivariable analyses, no statistically significant associations were found between KAP-based categories and trainee characteristics. Although the association did not reach statistical significance, participants who had received formal AI training were more likely to demonstrate high KAP-based readiness than those who had not received formal AI training (3/22, 13.6% vs 9/196, 4.6%). Similarly, participants supporting postgraduate AI training were more likely to have high KAP-based readiness than those who did not support AI training or were unsure (3/22, 6.9% vs 9/196, 1.7%; Table 4).

**Table 4.** Bivariable analysis of participants’ characteristics across the study-specific knowledge, attitude, and perception (KAP)–based readiness categories (N=218).

Variables and categories	Low KAP, n (%)	Moderate KAP, n (%)	High KAP, n (%)	*P* value
Age group (years)				.99
<30	13 (8.4)	133 (85.8)	9 (5.8)	
≥30	5 (7.9)	55 (87.3)	3 (4.8)	
Gender				.67
Man	2 (5.3)	35 (92.1)	1 (2.6)	
Woman	16 (8.9)	153 (85)	11 (6.1)	
Residency year				.96
Junior resident (years 1‐2)	10 (8.5)	100 (85.5)	7 (6)	
Senior resident (years 3‐5)	8 (7.9)	88 (87.1)	5 (5)	
Years since graduation				.87
Fresh graduate (<3 years)	8 (8.9)	78 (86.7)	4 (4.4)	
Senior graduate (≥3 years)	10 (7.8)	110 (85.9)	8 (6.3)	
Place of training				.07
Public hospital	8 (5.4)	130 (87.8)	10 (6.8)	
Private hospital	10 (14.3)	58 (82.9)	2 (2.9)	
Self-reported IT knowledge				.08
Below average or poorer	0 (0)	34 (97.1)	1 (2.9)	
Average or better	18 (9.8)	154 (84.2)	11 (6)	
Knowledge source of AI medical applications				.4
Internet	16 (9.7)	139 (84.2)	10 (6.1)	
Noninternet	2 (3.8)	49 (92.5)	2 (3.8)	
Had formal AI training during residency				.2
Yes	1 (4.5)	18 (81.8)	3 (13.6)	
No	17 (8.7)	170 (86.7)	9 (4.6)	
Belief that AI should be included in undergraduate training				.28
Yes	10 (6.6)	131 (86.8)	10 (6.6)	
No or not sure	8 (11.9)	57 (85.1)	2 (3)	
Belief that AI should be included in postgraduate training				.10
Yes	10 (6.3)	139 (86.9)	11 (6.9)	
No or not sure	8 (13.8)	49 (84.5)	1 (1.7)	

The multivariable linear regression model was not statistically significant (*F*_7,210_=1.049; *P*=.39; *R*²=0.034; adjusted *R*²=0.002). None of the participant characteristics included in the analysis was associated with the study-specific KAP-based readiness score. Two sensitivity analyses were conducted. In the first analysis, trainees’ beliefs about incorporating AI training into undergraduate and postgraduate medical education were added to the model, which improved the model fit (Δ*R*²=0.104; Δ*F*_2,208_=12.573; *P*<.001; *R*²=0.138). In the second analysis, years since graduation was added to the primary model, but this did not improve the model fit (Δ*R*²=0.001; Δ*F*_1,209_=0.136; *P*=.71). Although not statistically significant, higher adjusted KAP-based readiness scores were observed among trainees who had previously received formal AI training, those who trained at public hospitals, and those who used noninternet sources as their source of AI knowledge (Table 5).

**Table 5.** Multivariable linear regression analysis of factors associated with the study-specific knowledge, attitude, and perception (KAP)–based readiness score.

Variables and categories	Adjusted B coefficient (95% CI)	*P* value
Age group (years)		
<30	Reference	—^a^
≥30	0.673 (–1.812 to 3.158)	.59
Gender		
Man	Reference	—
Woman	0.324 (–2.577 to 3.224)	.83
Residency year		
Junior resident (years 1‐2)	Reference	—
Senior resident (years 3‐5)	–0.040 (–2.056 to 1.976)	.97
Place of training		
Public hospital	Reference	—
Private hospital	–1.664 (–3.903 to 0.574)	.14
Self-reported IT knowledge		
Below average or poorer	Reference	—
Average or better	–0.238 (–2.944 to 2.468)	.86
Source of AI knowledge		
Internet	Reference	—
Noninternet	1.723 (–0.617 to 4.063)	.15
Formal AI training during residency		
Yes	Reference	—
No	–2.507 (–5.827 to 0.812)	.14

a Not applicable.

## Discussion

### Main Findings

The participants showed moderate levels of AI-related knowledge, positive attitudes and perceptions, and moderate KAP-based readiness. Additionally, formal training in the medical applications of AI during residency was uncommon. The internet was the most frequently reported source of knowledge about the medical application of AI, and the majority of participants supported incorporating AI training into undergraduate and postgraduate medical education. No statistically significant associations were identified between study-specific KAP-based readiness categories and participants’ characteristics. Additionally, multivariable linear regression did not identify independent predictors of the total KAP-based readiness scores.

### Comparison With Prior Work

Readiness for the introduction of AI technologies to clinical practice includes factors related to health care professionals and health care facilities [25,26]. Therefore, successful implementation requires adequate knowledge and positive attitudes toward AI, which may encourage greater engagement with this emerging technology. Moreover, it requires supportive organizational infrastructure and policies [27,28]. Therefore, the KAP-based readiness scores in this study are part of the overall readiness evaluation.

The results showed that participants have moderate self-reported knowledge and moderately positive perception and attitude. While this may indicate that O&G trainees are generally aware of the technology, these results should not be considered as evidence of satisfactory readiness because discrepancies exist between O&G trainees’ self-reported IT and AI skills and their actual interaction with the AI technology in clinical scenarios [10], highlighting the need for structured training to improve readiness.

The significant positive correlations between self-reported knowledge, attitude toward the introduction of AI, and its perceived importance suggest that participants who reported greater self-reported knowledge tend to report higher positive attitude and perception scores (*P*<.001). While a similar pattern has been reported by another study [29], greater knowledge in the medical applications of AI may not translate to more positive attitudes, probably because health care professionals with greater knowledge of AI may have more awareness of its ethical and privacy-related implications, which may influence their attitudes toward its use [30].

This study showed that most of the participants never received formal training on the medical applications of AI. An earlier report that included internal medicine trainees showed similar results [31]. Additionally, three-quarters of the participants reported that the internet was their main source of knowledge about the medical application of AI. These findings are supported by the results of an earlier report that included medical and dental students [32]. Both findings are relevant and may be interrelated, as the lack of formal training may encourage participants to seek knowledge from informal sources, which may not always be reliable [33]. Moreover, structured training in the medical applications of AI was shown to significantly improve clinical competencies in diagnosis and treatment [34]. Additionally, formal AI knowledge sources such as conferences and scientific literature showed a positive association with better AI knowledge and attitudes [35]. Therefore, the limited formal training in medical AI technologies and reliance on informal sources of knowledge observed in this study highlight an important gap in current O&G training.

The participants in this study supported the integration of AI training into undergraduate and postgraduate medical education. Adding these two variables has improved the model in the sensitivity analysis. However, this improvement should be interpreted with caution, as these two variables are conceptually related to the attitude component of the KAP-based readiness score. Similar educational gaps and support have been reported by previous studies [18,31]. Moreover, limited formal AI training among medical students was associated with lower knowledge and less favorable attitudes toward its introduction [36]. Therefore, incorporating training on the medical applications of AI into undergraduate and postgraduate medical education may improve knowledge and enhance readiness for the introduction of AI technologies.

The current place of training in this study was not significantly associated with KAP-based readiness. However, low readiness was more frequently observed among trainees who trained at private hospitals. Differences in KAP-based readiness across training settings may be related to variations in organizational infrastructure and initiatives to introduce new technologies into clinical practice [37].

In this study, none of the measured trainees’ characteristics were significantly associated with KAP-based readiness. Therefore, further research should examine individual and organizational variables that may contribute to readiness, such as IT and AI literacy and training, health care professionals’ acceptance of the new technology, ethical concerns, and organizational infrastructure [38,39].

Assessing trainees’ knowledge, attitude, and perception before implementing structured training may identify gaps that should be addressed prior to introducing IT to clinical practice. The findings of this study contribute to the limited literature on O&G trainees’ perspectives toward AI and support the need for structured AI training to improve readiness.

### Strengths

This study specifically reported on the perspective of O&G trainees. The sample size was calculated in advance based on the available national number of trainees. Trainees were recruited from public and private hospitals, from different residency years, and from different geographical areas, therefore providing views from a wide range of training settings. The study assessed several related domains, including self-reported knowledge, attitude, perception, and KAP-based readiness. In addition, the questionnaire showed acceptable internal consistency across the different domains.

### Limitations

This study is cross-sectional; therefore, causal relationships between trainees’ characteristics and KAP-based readiness cannot be established. The questionnaire was developed specifically for this study and, therefore, was study-specific and not externally validated. The combined KAP-based readiness score was influenced more by the attitude domain because the 3 domains contained unequal numbers of items. Therefore, the score should not be interpreted as assigning equal weight to the 3 domains. Because of substantial missing item-level data in the excluded questionnaires, we were unable to compare them with the included questionnaires. IT and AI knowledge were not objectively assessed because this was not an aim of the study. Recruitment was based on trainees who were accessible at the time of data collection in the participating hospitals. The small proportion of item-level missing data was imputed, which may have negative impacts on the results.

### Conclusions

O&G trainees in Jordan demonstrated moderate self-reported AI-related knowledge, generally positive attitudes and perceptions of its importance, and moderate study-specific KAP-based readiness. Moreover, formal training on AI medical applications was uncommon, and most trainees supported the integration of AI training into medical education. These findings support the need for structured AI education, and future research should evaluate broader and objectively measured individual and organizational determinants of AI-related readiness.

## Supplementary material

10.2196/97072 Multimedia Appendix 1 Questionnaire.

## Abbreviations

KAP: knowledge, attitude, and perception
O&G: obstetrics and gynecology
STROBE: Strengthening the Reporting of Observational Studies in Epidemiology
