# Supplementary material for: Self-Reported Knowledge, Attitudes, Perceptions, and Readiness Regarding AI Among Obstetrics and Gynecology Trainees: Cross-Sectional Study
Source: JMIR Form Res. 2026 Sep 10;10:e97072. doi: 10.2196/97072 (PMC13561041; doi:10.2196/97072)
Supplement: Supplementary file 1 [file formative-v10-e97072-s001.docx]

**Knowledge, Attitudes, Perceptions, and Readiness Regarding Artificial Intelligence Among Obstetrics and Gynaecology Trainees in Jordan: A Cross-Sectional Study**

**Questionnaire Number:**

**1. Characteristics**

- **Age (years):**
- **Sex:** M	F
- **Residency year:** 1	2	3	4	5
- **Number of years since** graduation from medical school:
- **Place of current training:**

Ministry of Health	University hospitals	Military	Private

- **How would you describe your current knowledge of information technology?**

Very poor	Below Average	Above Average	Excellent

- **How would you rate your current understanding of the role of AI in O&G?**

Very poor	Below Average	Above Average	Excellent

- **How would you describe your current knowledge about the applications of AI in medicine?**

Very poor	Below Average	Above Average	Excellent

- **Source of information about applications of AI in medical practice?**

Local lecture	Conference presentation	Media	A colleague

- **Are you willing to use AI in clinical practice?**

Never	Seldom	Some of the times	Most of the times

- **Have you had formal training about the applications of AI during residency training?**

Yes	No

- **How important do you think AI could be in your area of work?**

Very Important	Important	Moderately Important	Slightly Important	Not Important

**2. Trainees Knowledge about the Application of AI in O&G**

**How much do you agree with the following statements regarding your knowledge about current applications of AI in O&G?**

- **AI can predict some pregnancy complications such as preterm labor.**

Strongly Agree	Agree	Undecided	Disagree	Strongly Disagree

- **AI contributes to a better interpretation of CTG.**

Strongly Agree	Agree	Undecided	Disagree	Strongly Disagree

- **AI aids in determining the duration and regularities of the menstrual cycles.**

Strongly Agree	Agree	Undecided	Disagree	Strongly Disagree

- **AI improves the timing of embryo transfer in IVF.**

Strongly Agree	Agree	Undecided	Disagree	Strongly Disagree

- **AI contributes to the analysis of pelvic ultrasound scan images.**

Strongly Agree	Agree	Undecided	Disagree	Strongly Disagree

- **AI can analyse pelvic ultrasound scan images to identify the presence of endometriosis.**

Strongly Agree	Agree	Undecided	Disagree	Strongly Disagree

- **AI can analyse pelvic ultrasound scan images and identify signs of ovarian cancer with** **high sensitivity.**

Strongly Agree	Agree	Undecided	Disagree	Strongly Disagree

- **AI can analyse histopathological images to identify the presence of cancer cells.**

Strongly Agree	Agree	Undecided	Disagree	Strongly Disagree

- **AI can predict the likelihood of a positive outcome following treatment of ovarian cancer.**

Strongly Agree	Agree	Undecided	Disagree	Strongly Disagree

**3. Trainees’ Attitude Towards the Introduction of AI** **in O&G**

**How much do you agree with the following statement?**

- **AI is relevant in current O&G practice.**

Strongly Agree	Agree	Undecided	Disagree Strongly Disagree

- **AI may improve the outcome of women’s healthcare.**

Strongly Agree	Agree	Undecided	Disagree	Strongly Disagree

- **O&G doctors do not know enough about AI to use it safely with patients.**

Strongly Agree	Agree	Undecided	Disagree	Strongly Disagree

- **There is enough public awareness of AI in our community.**

Strongly Agree	Agree	Undecided	Disagree	Strongly Disagree

- **Doctors have become heavily dependent on computer systems.**

Strongly Agree	Agree	Undecided	Disagree	Strongly Disagree

- **The use of AI interferes with doctor’s learning curve and therefore may negatively affect doctors’ abilities in the future to make correct judgments about patient management. (1)**

Strongly Agree	Agree	Undecided	Disagree	Strongly Disagree

- **Al testing must be performed before use on patients by an independent body (such as a health authority). (1)**

Strongly Agree	Agree	Undecided	Disagree	Strongly Disagree

- **The use of Al impairs the physician-patient relationship.**

Strongly Agree	Agree	Undecided	Disagree	Strongly Disagree

- **AI helps patients feel less embarrassed when talking about sensitive symptoms with their doctor.**

Strongly Agree	Agree	Undecided	Disagree	Strongly Disagree

- **AI contributes to maintaining the privacy and confidentiality of patient-doctor interactions and medical procedures.**

Strongly Agree	Agree	Undecided	Disagree	Strongly Disagree

- **The implementation of AI in clinical practice is a huge burden for patients and medical staff.**

Strongly Agree	Agree	Undecided	Disagree	Strongly Disagree

- **AI will dominate O&G clinical practice in the next few years.**

Strongly Agree	Agree	Undecided	Disagree	Strongly Disagree

- **AI should be part of the O&G training programs.**

Strongly Agree	Agree	Undecided	Disagree	Strongly Disagree

- **AI will improve healthcare particularly where experts are not available.**

Strongly Agree	Agree	Undecided	Disagree	Strongly Disagree

- **Using Al will give doctors more time to spend with patients.**

Strongly Agree	Agree	Undecided	Disagree	Strongly Disagree

- **The use of Al is changing the demands of the medical profession.**

Strongly Agree	Agree	Undecided	Disagree	Strongly Disagree

- **AI will help analyse patient medical data and offer the most appropriate treatment.**

Strongly Agree	Agree	Undecided	Disagree	Strongly Disagree

- **If Al can predict a low chance of a patient's survival, doctors may not fight for that patient's life as much as before.** Strongly Agree	Agree	Undecided	Disagree	Strongly Disagree
- **If a patient is harmed from NOT using AI, the physician must take responsibility for not following Al's recommendations.**

Strongly Agree	Agree	Undecided	Disagree	Strongly Disagree

- **AI can contribute to improving maternal healthcare and pregnancy outcomes.**

Strongly Agree	Agree	Undecided	Disagree	Strongly Disagree

- **AI can play a role in empowering women to make informed decisions about their sexual and reproductive health.**

Strongly Agree	Agree	Undecided	Disagree	Strongly Disagree

**4. Trainees’ Perception of the Importance of AI**

**How much do you agree with the following statements regarding your perception of the importance of AI applications in current medical practice?**

- **Integration of AI in O&G can contribute to reducing healthcare costs.**

Strongly Agree	Agree	Undecided	Disagree	Strongly Disagree

- **AI will improve the efficiency and organization of care in O&G.**

Strongly Agree	Agree	Undecided	Disagree	Strongly Disagree

- **AI could make the most significant impact in specific areas within O&G.**

Strongly Agree	Agree	Undecided	Disagree	Strongly Disagree

- **AI helps in reducing the rates of misdiagnoses.**

Strongly Agree	Agree	Undecided	Disagree	Strongly Disagree

- **The use of Al may be considered an effective instrument against the overload of physicians and the shortage of physicians. (1)**

Strongly Agree	Agree	Undecided	Disagree	Strongly Disagree

- **AI will make doctors play a less important role in treating patients in the future.**

Strongly Agree	Agree	Undecided	Disagree	Strongly Disagree
